# Supplementary material for: Prediction of radiation pneumonitis using dose-volume histogram parameters with high attenuation in two types of cancer: A retrospective study
Source: PLoS One. 2020 Dec 28;15(12):e0244143. doi: 10.1371/journal.pone.0244143 (PMC7769248; doi:10.1371/journal.pone.0244143)
Supplement: S1 Table — (DOCX) [file pone.0244143.s001.docx]

S1 Table. The following dosimetric parameters were evaluated.

| Parameter |  |
| --- | --- |
| HAV (cc) | The volume of high attenuation lung |
| MLD (Gy) | Mean lung dose |
| MHALD (Gy) | Mean high attenuation lung dose |
| HAV2 (cc) | The volume of the high attenuation lung receiving a dose of ≥ 2 Gy |
| HAV5 (cc) | The volume of the high attenuation lung receiving a dose of ≥ 5 Gy |
| HAV10 (cc) | The volume of the high attenuation lung receiving a dose of ≥ 10 Gy |
| HAV20 (cc) | The volume of the high attenuation lung receiving a dose of ≥ 20 Gy |
| HAV30 (cc) | The volume of the high attenuation lung receiving a dose of ≥ 30 Gy |
| V2 | The percentage of lung volume receiving ≥ 2 Gy to total lung volume |
| V5 | The percentage of lung volume receiving ≥ 5 Gy to total lung volume |
| V10 | The percentage of lung volume receiving ≥ 10 Gy to total lung volume |
| V20 | The percentage of lung volume receiving ≥ 20 Gy to total lung volume |
| V30 | The percentage of lung volume receiving ≥ 30 Gy to total lung volume |
| HAV2% | The percentage of high attenuation lung volume receiving ≥ 2 Gy to total lung volume |
| HAV5% | The percentage of high attenuation lung volume receiving ≥ 5 Gy to total lung volume |
| HAV10% | The percentage of high attenuation lung volume receiving ≥ 10 Gy to total lung volume |
| HAV20% | The percentage of high attenuation lung volume receiving ≥ 20 Gy to total lung volume |
| HAV30% | The percentage of high attenuation lung volume receiving ≥ 30 Gy to total lung volume |
| HAV2 ∕ HAV | The ratio of the high attenuation lung volume receiving ≥ 2 Gy to total high attenuation lung volume |
| HAV5 ∕ HAV | The ratio of the high attenuation lung volume receiving ≥ 5 Gy to total high attenuation lung volume |
| HAV10 ∕ HAV | The ratio of the high attenuation lung volume receiving ≥ 10 Gy to total high attenuation lung volume |
| HAV20 ∕ HAV | The ratio of the high attenuation lung volume receiving ≥ 20 Gy to total high attenuation lung volume |
| HAV30 ∕ HAV | The ratio of the high attenuation lung volume receiving ≥ 30 Gy to total high attenuation lung volume |
