# Supplementary material for: Prediction of radiation pneumonitis using dose-volume histogram parameters with high attenuation in two types of cancer: A retrospective study
Source: PLoS One. 2020 Dec 28;15(12):e0244143. doi: 10.1371/journal.pone.0244143 (PMC7769248; doi:10.1371/journal.pone.0244143)
Supplement: S2 Table — (DOCX) [file pone.0244143.s002.docx]

S2 Table. Chemotherapy regimens used in the study.

Lung cancer

| Timing of Administration | Chemotherapeutic Agents | No. of Patients |
| --- | --- | --- |
| Prior to radiotherapy | Docetaxel | 1 |
|  | Vinorelbine | 2 |
|  | Carboplatin and nab-paclitaxel | 1 |
| Concurrent with radiotherapy | Carboplatin and paclitaxel | 14 |
|  | Cisplatin and etoposide | 7 |
|  | Nedaplatin and docetaxel | 7 |
|  | Cisplatin and vinorelbine | 5 |
|  | Carboplatin and etoposide | 5 |
|  | Cisplatin and docetaxel | 1 |

Esophageal cancer

| Timing of Administration | Chemotherapeutic Agents | No. of Patients |
| --- | --- | --- |
| Concurrent with radiotherapy | Cisplatin and 5-Fluorouracil | 62 |
|  | tegafur/gimeracil/oteracil | 1 |
|  | Leucovorin and 5-Fluorouracil | 1 |
