# Supplementary material for: Prediction of radiation pneumonitis using dose-volume histogram parameters with high attenuation in two types of cancer: A retrospective study
Source: PLoS One. 2020 Dec 28;15(12):e0244143. doi: 10.1371/journal.pone.0244143 (PMC7769248; doi:10.1371/journal.pone.0244143)
Supplement: S3 Table — (DOCX) [file pone.0244143.s003.docx]

S3 Table. Univariate logistic regression analysis of dosimetric parameters for symptomatic radiation pneumonitis (≥ Grade 2) in lung cancer and esophageal cancer

| Parameter | P-value  (Lung cancer) | P-value  (Esophageal cancer) |
| --- | --- | --- |
| V2 | <0.0001 | 0.0930 |
| V5 | <0.0001 | 0.0023 |
| V10 | <0.0001 | 0.0004 |
| V20 | <0.0001 | 0.0061 |
| V30 | <0.0001 | 0.0011 |
| HAV2% | <0.0001 | 0.0013 |
| HAV5% | <0.0001 | 0.0004 |
| HAV10% | <0.0001 | <0.0001 |
| HAV20% | <0.0001 | 0.0002 |
| HAV30% | <0.0001 | 0.0004 |
| HAV2 ∕ HAV | <0.0001 | 0.0089 |
| HAV5 ∕ HAV | <0.0001 | 0.0026 |
| HAV10 ∕ HAV | <0.0001 | 0.0005 |
| HAV20 ∕ HAV | <0.0001 | 0.0073 |
| HAV30 ∕ HAV | <0.0001 | 0.0015 |
| MLD (Gy) | <0.0001 | 0.0005 |
| MHALD (Gy) | <0.0001 | 0.0007 |

V2/5/10/20/30 = percentage of lung volume irradiated with ≥ 2/5/10/20/30 Gy, respectively; HAV2/5/10/20/30% = percentage of the lung with high attenuation volume (HAV) receiving 2/5/10/20/30 Gy respectively; HAV2/5/10/20/30 = volume of the lung with high attenuation volume (HAV) receiving 2/5/10/20/30 Gy, respectively; HAV= lung with high attenuation volume; MLD = mean lung dose; MHALD = mean high attenuation lung dose.
