# Supplementary figures and images for: Prediction of radiation pneumonitis using dose-volume histogram parameters with high attenuation in two types of cancer: A retrospective study
Source: PLoS One. 2020 Dec 28;15(12):e0244143. doi: 10.1371/journal.pone.0244143 (PMC7769248; doi:10.1371/journal.pone.0244143)

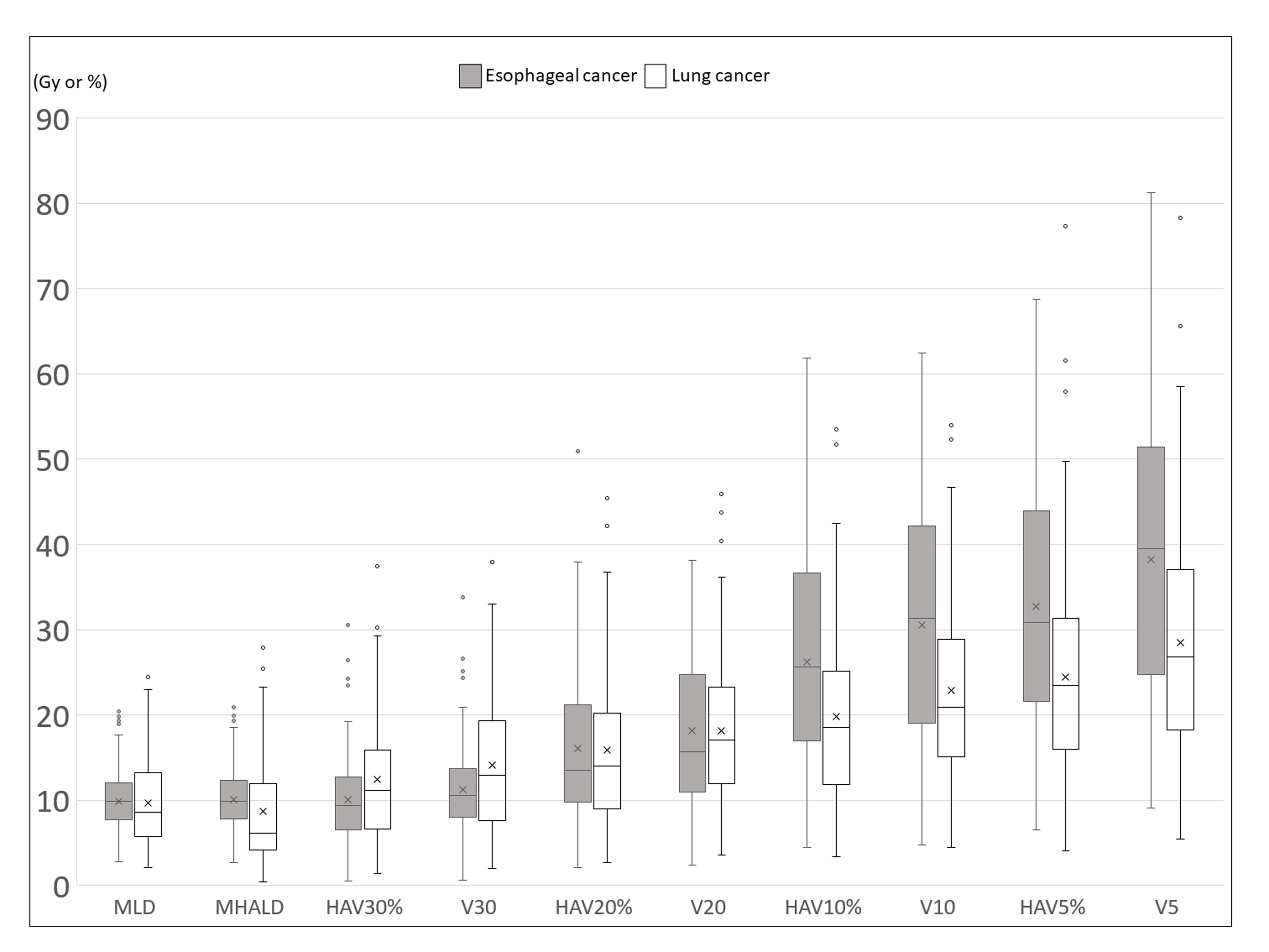

Supplement: S1 Fig — (TIF) [file pone.0244143.s004.TIF]
