# Supplementary material for: Prediction of radiation pneumonitis using dose-volume histogram parameters with high attenuation in two types of cancer: A retrospective study
Source: PLoS One. 2020 Dec 28;15(12):e0244143. doi: 10.1371/journal.pone.0244143 (PMC7769248; doi:10.1371/journal.pone.0244143)
Supplement: Attachment — Submitted filename: Response to Reviewers.docx [file pone.0244143.s005.docx]

**Response to Reviewer #1:**

Thank you very much for providing us with important insights. We are delighted to hear that you think our work will spark debate in our field. In the following sections, you will find our responses to each of your points and suggestions. We are grateful for the time and energy you expended in reviewing this work.

Major things

Choice of CT number

Please expand on the choice of -856 HU, why this value? could other choices be made?
Ideally one would perform your LAV segmented analysis for a range HU and find the HU number cut-off with the largest improvement in over the non-segmented metrics.

**Response:**

In general, most studies on COPD have used a CT cut-off value of -950 HU for inspiration and -856 HU for expiration as the cut-off value for emphysematous lesions. Because the regions suggesting an emphysematous lesion in the free-breathing CT images used in this study is close to the images taken during expiration, we used -856HU, which is commonly used during expiration.
To further verify the validity of this cutoff value, we previously validated the association between CT under free breathing and the inspiratory CT performed within 45 days after free breathing CT. Inspiratory CT was performed using Toshiba Aquillion ONE (Toshiba Medical Systems Corp., Otawara, Tochigi, Japan) and LAV was analyzed using the Aquarius iNtuitionTM software ver.4.4.12 (TeraRecon Inc., San Mateo, Calif) and evaluated using the threshold limit of -950 HU. We found that the LAV in inspiratory CT was highly correlated with the LAV in CT under free breathing. This detail can be found in reference #9. We've added these details on lines 87-91.

Direct comparison of the of new segmented HAV metrics to corresponding traditional V metrics
The data suggests that the percentage of low attenuation volume is roughly 10% or less of the total lung volume. Doesn’t this mean that the segmentation is only a small (roughly 10% or less) correction compared to standard analyses without the LAV segmentation?

**Response:**

This correction may seem trivial. However, there were differences between individual patients, even if the overall rate was 10%. Some patients had more emphysematous lesions and others had less, and differences appeared between those with larger and smaller corrections.

An error in the unit of values for the lung cancer population has been corrected.

Please directly compare and comment on the statistical significance of the differences between segmented and non-segmented metrics g.g. HAV20% = 33.7 and V20% = 35.2 for lung cancer. Total patients is 77. Is this segmented HAV20% really a significant improvement on the V20%? My quick visual comparison suggests that most the “V” metrics give very similar results to their HAV counterparts.

Same also for the ROC results: e.g HAV10% = 0.869 vs V10%=0.849 in the esophageal ROC figure. Is this difference significant?

**Response:**

We agree that you are making a fair point. The AUC was used as a measure of accuracy in this study. In our previous study, we have assessed the AUC, as well as other indices such as NRI (net reclassification improvement) and IDI (integrated discrimination improvement). Although we did not present significant differences in individual values, the main purpose of this study was to compare cut-off values from a highly accurate index to ascertain universality. The purpose was not to compare significant differences in individual values.

Important missing technical details

If possible, also give the mass density or electron densities that the -856 HU threshold value corresponds to for your CT scanner (or at least give a density range).

**Response:**

Regarding relative electron density, −856HU was 0.1488. We have added these details on lines 92-93.

Please specify the dose calculation algorithms used? This may be particularly significant as simpler algorithms such as Varian’s AAA algorithm, is known to overestimate dose to low density lung volumes compared to Boltsmann-solver or Monte-Carlo algorithms such as accuros.

**Response:**

The treatment plans were designed in the Eclipse treatment planning system by the correction-based algorithm AAA (version 13.6.23) and the calculation grid was 2.5 mm for the lung. (lines 80-81)

Please specify the breathing phase of the CT scan (inspiration breathhold , free breathing,4$D CT at a given phase etc). Note that you may want to add to the discussion that for inspiration-breathold scans which are commonly used for SBRT lung radiotherapy treatments would have larger LAV% and hence larger differential between the segmented and non-segmented metrics.

**Response:**

We have added it on line 84 and 244-246.

Please also discuss the margins and motion management during treatment (presumably no gating, no breathold was used on these treatments?).

**Response:**

Gating and breath-hold were not used, as you pointed out. We have included this information in the manuscript.

Smaller things

line 49
“have set vague cut-off values for”
strongly suggest to remove word “vague” as the metrics themselves are not vague.

Use of “cut-off” is unusual. Suggest to replace “cut-off” with “constraints” as the constraints can be one of a number of metrics such as: volume that gets at least a given dose, mean dose, max dose.

**Response:**

We have removed the word “vague” and replaced “cut-off” with “constraints.”

Line 50-51
“lung volume receiving 20 Gy or more (V20%) (30−40%) to reduce the risk of RP”

Terminology as given is confusing: We need both Dose in Gy and volume in % to define the “V” metrics. Normally the first number (eg V20) is the dose in Gy, not % dose nor % volume. E.g. V20 < 30% means the volume that receives at least 20 Gy should be less than 30% of the lung volume. Here by writing V20% you are implying that the 20 refers to a %, where it normally refers to the dose in Gy. Please correct the your “V20%” notation to the more standard notation “V20 <20%” and complete notation defining both the dose and the volume.

For example see the RTOG 0937 protocol where this constraint originated: https://www.rtog.org/ClinicalTrials/ProtocolTable/StudyDetails.aspx?action=openFile&FileID=13697

**Response:**

Thank you for your comment. We have removed "%."

Line 52
For clarity suggest to explain or expand on “low attenuation” and “high attenuation” straightaway eg consider “volumes of low attenuation in CT imaging” Suggest to also apply to abstract.

**Response:**

Thank you for pointing this out. Your recommendation has been followed, the abstract inclusive.

Line 52
It is technically wrong to say “areas of low attenuation” you mean “volumes of low attenuation”

**Response:**

We have replaced with "volumes," as suggested.

Line 80
“using the threshold limit of −856 HU”
Replace with
“using the upper threshold limit of −856 HU”

**Response:**

Following your recommendation, we have added "upper" to the phrase.

Line 106 please specify more clearly and consistent with standard notation
Replace “V2%, V5%, V10%, V20%, and V30%,”

with presumed meaning: “V20 Gy < 2%, 5%, 10%, 20%, 30%”

Please also clearly specify that it is the volume of both lungs if that is the case.

**Response:**

Thank you for your comment. We have removed "%."

Tables
I find the extreme-spaced tables impossible to digest. E.g table 1 is only 3 data columns but spans 3 pages.
data too spread out to meaningfully digest.

**Response:**

Thank you for your comment. We have revised the tables for clarity.

Please specify the resolution, or at least range of resolutions / slice thickness settings used for the CT scans. E.g. voxel size can effect the segmented volumes if the resolution is too course. e.g a voxel that is 4/5 with air 1/5 dense tumor tissue will represented by approx -800HU

**Response:**

Thank you for your comment. We have included this information on lines 82-82.

Reviewer #2: The manuscript was well written and the analysis was properly done to support the conclusion.
There are a few suggestions for author to consider:

1. It's not very clear in the manuscript how the MU threshold of “- 856” was determined. Was there any systematic analysis done? As you have presented in the manuscript, by setting such a threshold it will impact the selection of optimal dose evaluation criteria and corresponding volume threshold. It's probably a good idea to address this issue to ensure that the difference in dose evaluation was not caused by the uncertainty of the chosen threshold.

**Response:**

In general, most studies on COPD have used a CT cut-off value of -950 HU for inspiration and -856 HU for expiration as the cut-off value for emphysematous lesions. Because the regions suggesting an emphysematous lesion in the free-breathing CT images used in this study is close to the images taken during expiration, we used -856HU, which is commonly used during expiration.
To further verify the validity of this cutoff value, we previously validated the association between CT under free breathing and the inspiratory CT performed within 45 days after free breathing CT. Inspiratory CT was performed using Toshiba Aquillion ONE (Toshiba Medical Systems Corp., Otawara, Tochigi, Japan) and LAV was analyzed using the Aquarius iNtuitionTM software ver.4.4.12 (TeraRecon Inc., San Mateo, Calif) and evaluated using the threshold limit of -950 HU. We found that the LAV in inspiratory CT was highly correlated with the LAV in CT under free breathing. This detail can be found in reference #9.

2. As what I have noticed that only a small portion of plans were done with IMRT technique, this can potentially introduce bias since static IMRT or VMAT plans can have higher, if not much higher, low dose contribution to the treatment. That means if the portion of the IMRT plan increases, one may see the optimal dose evaluation criteria shift towards low dose.

**Response:**

Thank you for pointing this out. We have included information regarding this on lines 246-249

3. For conventional lung radiation therapy, I can imagine the fractional dose would be similar. However, it would be clearer to include the fractional dose information rather than just the total dose > 30Gy when demonstrating the patient selection criteria.

Response:

The fraction dose is 1.8–3.0 Gy. We have included this in the manuscript on line 71.
