# Supplementary material for: Prediction of radiation pneumonitis using dose-volume histogram parameters with high attenuation in two types of cancer: A retrospective study
Source: PLoS One. 2020 Dec 28;15(12):e0244143. doi: 10.1371/journal.pone.0244143 (PMC7769248; doi:10.1371/journal.pone.0244143)
Supplement: Attachment — Submitted filename: Response to Reviwers.docx [file pone.0244143.s006.docx]

**Response to Reviewer #2:**

Thank you very much for providing us with important insights. We are delighted to hear that you think our work will spark debate in our field. In the following sections, you will find our responses to each of your points and suggestions. We are grateful for the time and energy you expended in reviewing this work.

Line 225 - This suggestion may not be appropriate for two reasons: 1. As you have mentioned HAV30% is not the most sensitive predictor of RP for esophagus cases, therefore it’s necessary to have multiple/other predictors in order to reach a useful sensitivity. 2. HAV30% is not the most sensitive predictor for a reason. It’s unlikely that

HAV30%/V30 from esophagus will be as high as that in lung cases simply because of the anatomies and beam arrangements (i.e. unlikely you will see an APPA treatment for esophagus as what you have shown as an example for lung). Therefore, the onset of RP could be dominated by the low dose to lung for esophagus cases, which was suggested by the shift of most sensitive predictors from HAV30% in lung to HAV10% and HAV5% in esophagus cases. Therefore, suggesting using HAV30% for both sites can be misleading and may not be clinically useful.

**Response:**

We deleted this sentence.

I would also suggest author to generate bar plots of each DVH parameter side by side for the two sites so that it’s clear how the distribution of those parameters different from each other, and compare the calculated thresholds with the 75% percentile of each parameter to see if using that specific predictor is meaningful. For example if the 75% percentile of

HAV30% for esophagus cases is 5% and the HAV30% threshold is 11%, it would not be useful to suggest HAV30% as a RP predictor for esophagus cases.

**Response:**

We created box plots instead of bar blots to clarify the 75% percentile (S4 Figure). As you pointed out, the 75% percentile of HAV20% and V20 and V5 in esophageal cancer were lower than threshold. Therefor this point was added as the Limitation to Discussion.
